# Book Review: Psicología de la Creatividad. Perspectivas Contemporáneas

**DOI:** 10.3389/fpsyg.2021.717767

**Published:** 2021-09-09

**Authors:** Vicente Alfonso-Benlliure

**Affiliations:** Department of Developmental and Educational Psychology, University of Valencia, Valencia, Spain

**Keywords:** psychology, divergent thinking, problem finding and solving, creative personality, creativity

This work is the reissue, two decades later, of a book that became a reference for the study of creativity in the Spanish language. The current, renewed, completed and updated edition maintains the rigor of the previous edition and its direct, personal, and lucid expressive style.

The book “Psicología de la Creatividad. Perspectivas contemporáneas” has an informative character and a scientific basis and offers an analytical approach to the study of creativity from the perspective of psychology, emphasizing the cognitive and motivational dimensions. This is a welcome reissue. It reviews the research literature over the past 12 years and offer an updated version of the state of the art in the field of creativity.

The book is divided into six large sections. The first, *The mystery of genius*, includes a series of chapters dedicated to unraveling the deep roots of the creative phenomenon, intermixed with implicit conceptions and mythical visions of artistic genius. With ease, the text dissects what is and what is not creativity, offering a more realistic, democratic (every person have creative potential than can be developed) and useful (both for creativity students and for education professionals) vision of creativity. Many other myths are unmasked and exposed in the light of a methodical and clarifying analysis: the insight or the “aha” experience, the role of chance in the creative process, the innate character of creativity, or genius in creative artists and scientists.

The second part, entitled *Creative products*, includes three chapters dedicated to how creative results can be considered as such: their basic characteristics, what criteria to use to assess them or the environmental influences. Without a product there is no creativity, “create is a transitive verb,” the author insists. Both, historical creativity that marks a watershed moment in a given field (e.g., Pythagoras' theorem, the Mona Lisa) and daily creativity (e.g., a tasty dish with what was left in the fridge, flower arranging…) requires that certain psychological processes lead to an idea or product that marks the culmination of the creative process, although it does not close it.

The third part (*Explanations of creativity*) addresses, from a theoretical perspective, the explanation of the multifaceted nature of creativity. It also reviews the concepts and procedures that underpin the creative process, as well as the need for sustained work to make it possible. The author carries out an interesting synthesis of the historical trajectory on the scientific study of creativity. She states that a complex reality must be analyzed from a multidisciplinary approach. This part of the book emphasizes the relevance of the component models of creativity, and among them, a personal commitment to cognitive and motivational components.

The fourth part (*The creative thinking*) is made up of three chapters: problem finding, insight and analogical thinking. The author states that what incites the creative process is “doubt,” that leads to a question and a problem to solve. People with developed creativity are familiar with self-discovered problems. When they have no problems, creative people look for them. These creative ideas can be achieved as a consequence of conscious and deliberate cognitive process, but also as a result of parallel and unconscious processing, like dreams, reveries or insight experiences.

The fifth part (*The reasons to create*) is dedicated to the motivational factors that sustain and direct the creative effort. These chapters review the theories of intrinsic motivation linked to creativity. They also review the personal motives that help to understand where that energy comes from. Among these personal motives, the author delves into conflictive territories like the role of achievement motives, self-realization, sublimation, and more spurious reasons such as ambition, reputation or narcissism. You can be creative out of “pure selfishness.”

The sixth and last part of the book (*Personality*) have been added in this edition. It addresses the personality characteristics of the most creative people. A historical journey through the studies of personality is made, paying special attention to the factor most solidly linked to creativity: openness to experience. Moreover, some other complementary explanations about personality traits (recurrently present in studies on eminent creativity) are included such as perseverance, risk tolerance and independence.

Finally, a debate, still controversial, is also openly addressed: does creativity have a dark side? Can psychological disorders promote creativity? To understand how, when and where “madness” can be creative, the question of the specificity of domains is reviewed. The myth of the madness associated with creativity only has a place in the context of art, in which the criterion of adequacy is laxer and ambiguous. Even in that context, the author concludes that there is no causal relationship, that the artist creates “despite madness.”

Throughout the work, the author adopts a clear theoretical position, advocating a way of understanding creativity in which the cognitive and motivational components play a special role. This positioning implies its main limitation: although at no time the influence of the affective-emotional components is denied, the author assumes that this influence is subordinate to others. Thus, it does not consider the role that positive and negative emotions have on the creative process. Neither, the creative effort as a way to manage and express emotions, or the existence of an “emotional creativity,” understood as the ability to experience and express original, appropriate and authentic combinations of emotions.

In summary, this is a necessary, updated and well-executed reissue, supported by rigorous scientific references and peppered with numerous philosophical reflections, references to specific artistic works, as well as varied anecdotes that enrich the reading. The complexity of the creative phenomenon is partly responsible for the existence, even today, of prejudices toward it from Psychology and some of its professionals. The author factually states that Psychology still does not pay sufficient attention to creativity, which for many, is the most complex dimension of human thinking. Publications like this, help to overcome some of those preconceptions and deepen the understanding of that evolutionary advantage of human beings called creativity.

## Author Contributions

The author confirms being the sole contributor of this work and has approved it for publication.

## Conflict of Interest

The author declares that the research was conducted in the absence of any commercial or financial relationships that could be construed as a potential conflict of interest.

## Publisher's Note

All claims expressed in this article are solely those of the authors and do not necessarily represent those of their affiliated organizations, or those of the publisher, the editors and the reviewers. Any product that may be evaluated in this article, or claim that may be made by its manufacturer, is not guaranteed or endorsed by the publisher.

